# Therapeutic Potential of Tuna Backbone Peptide and Its Analogs: An In Vitro and In Silico Study

**DOI:** 10.3390/molecules26072064

**Published:** 2021-04-03

**Authors:** Varun Gopinatth, Rufa L. Mendez, Elaine Ballinger, Jung Yeon Kwon

**Affiliations:** 1 Department of Integrative Biology, Oregon State University, Corvallis, OR 97331, USA; gopinatv@oregonstate.edu; 2 Department of Food Science and Technology, Oregon State University, Corvallis, OR 97331, USA; mendezru@oregonstate.edu (R.L.M.); elaine.ballinger@oregonstate.edu (E.B.); 3 Seafood Research and Education Center, Oregon State University, Astoria, OR 97103, USA

**Keywords:** bioactive peptide, in vitro, in silico, multifunctional, seafood by-product

## Abstract

Tuna backbone peptide (TBP) has been reported to exert potent inhibitory activity against lipid peroxidation in vitro. Since this bears relevant physiological implications, this study was undertaken to assess the impact of peptide modifications on its bioactivity and other therapeutic potential using in vitro and in silico approach. Some TBP analogs, despite lower purity than the parent peptide, exerted promising antioxidant activities in vitro demonstrated by ABTS radical scavenging assay and cellular antioxidant activity assay. In silico digestion of the peptides resulted in the generation of antioxidant, angiotensin-converting enzyme (ACE), and dipeptidyl peptidase-IV (DPPIV) inhibitory dipeptides. Using bioinformatics platforms, we found five stable TBP analogs that hold therapeutic potential with their predicted multifunctionality, stability, non-toxicity, and low bitterness intensity. This work shows how screening and prospecting for bioactive peptides can be improved with the use of in vitro and in silico approaches.

## 1. Introduction

Biologically active proteins and peptides have been an expanding research area for the past decades, owing to their promising therapeutic potential and industry applications [1,2,3]. The growing awareness for their health-promoting effects drives the significant expansion of its share in the functional food and nutraceutical market, despite major production and validation bottlenecks [4,5,6,7]. Food-derived bioactive peptides (BAPs) have been gaining attention as a therapeutic option for lifestyle-related diseases like obesity, Type 2 diabetes, and cardiovascular diseases [8], with their high potency, selectivity, coupled with low toxicity reports [9]. Peptides from terrestrial plants and animals as well as aquatic sources have been reported to favorably affect the immune (antimicrobial, immunomodulatory, and anti-cancer), cardiovascular (antithrombotic, antidiabetic, antihypertensive, hypolipidemic, anti-inflammatory), nervous (antinociceptive, relaxing, anti-amnesic), and gastrointestinal (anti-obesity) health [10].

Marine protein hydrolysates and BAPs are of interest since species diversity and highly variable growth conditions may give rise to protein precursors that are different from terrestrial sources. While the range of potential applications may be comparable to terrestrial materials [6,11,12], it is the growing effort on seafood valorization that further intensifies BAP recovery from seafood sources. With the growing demand for seafood-based products, processing plants discard a significant amount of protein-rich waste that can be used for the recovery of biologically active and functional biomaterials [13,14,15]. Numerous bioactivities have been reported in hydrolysates and peptides from fish processing by-products like trimmings, skins, heads, backbones and frames, viscera, even wastewater [15,16,17]. 

Antioxidant peptides constitute a large portion of the data library for aquatic material-sourced BAPs. Peptides from the skin of thornback ray [18], leatherjacket [19,20] giant catfish [21], croaker and horse mackerel [22], Pacific cod [23], skate [24], seabass [25], tilapia [26], grass carp [27] as well as the bony structures like pectoral fin of salmon [28], frame from tilapia [29] and backbones of salmon [30,31], barracuda and ribbon fish [32], mackerel [33], and tuna [34] have shown promising antioxidant activities. Furthermore, numerous seafood-derived antioxidant peptides exhibited multifunctionalities like angiotensin-converting enzyme (ACE) [23,24,35,36,37] and dipeptidyl peptidase (DPPIV) enzyme inhibition [36,37], immunomodulatory [38,39,40,41], antifatigue [26], even wound-healing [42] properties. Since most of the lifestyle-related disease pathologies are associated with oxidative stress [43,44], antioxidant peptides that can stimulate better physiological stress response, while exerting other preferred bioactivities, hold promise for health improvement [7,45].

While there are numerous research works on BAPs, very few reach commercialization due to challenges relating to high production cost, bioavailability and bio-efficacy, bitterness, and potential toxicity [5,10]. Since the traditional approach for BAP research employs the determination of precursor protein and hydrolytic conditions to generate potential bioactive products that are assayed against different bioactivity screening models, this part alone consumes a significant amount of time and financial resources. On top of that, some in vitro models are challenged by their translational value and physiological relevance, as in the case of some antioxidant assays [46,47]. To overcome these challenges, there has been an increasing number of bioinformatics-based tools and platforms to help determine suitable protein sources and hydrolytic conditions, identify/predict BAPs generated, and predict numerous physicochemical properties that may have a significant impact on bioactivity, bioaccessibility, stability, toxicity, and sensory acceptability [48,49]. This not only helps reduce the time needed to shortlist the most promising BAPs from a sample batch of a much larger size, but it also helps save the limited financial resource to funding more targeted assays for validation. Although the cost may remain relatively high for BAP products, the wider applicability for multifunctional peptides can help make to cost more acceptable [50]. 

For this study, we used tuna backbone peptide (TBP) [34] to investigate how specific peptide sequence modifications can affect its antioxidant property, as well as its ability to remain bioactive after in silico digestion. Since most antioxidant BAPs have the potential to be multifunctional, we predicted other potential bioactivities for TBP and its analogs using in silico platforms. We assessed the usability of the identified multifunctional BAPs in terms of peptide stability, toxicity, and bitterness in silico. With this investigation, we show how an antioxidant peptide can be a promising therapeutic option for health improvement using in vitro and in silico approaches.

## 2. Results

### 2.1. Peptide Modification Affects Antioxidant Activities

**Figure 1:**
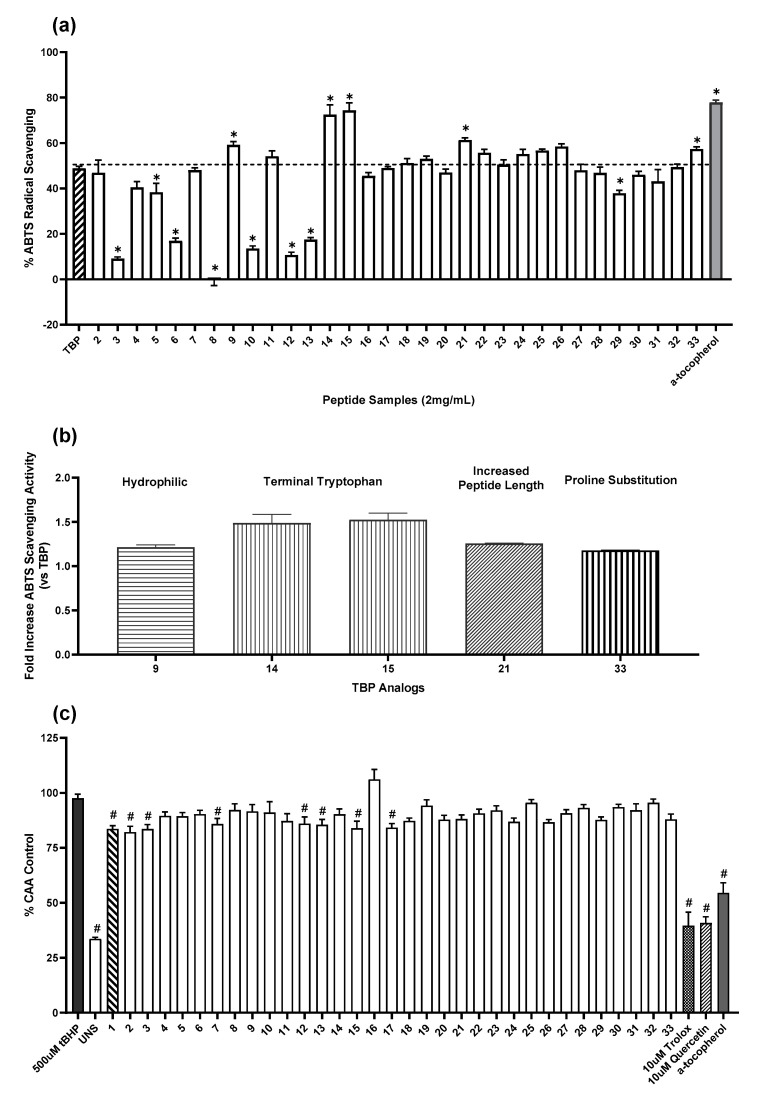
Antioxidant activities of tuna backbone peptide (TBP) and its analogs were affected by peptide modification using (**a**,**b**) ABTS Radical Scavenging Assay and (**c**) Cellular Antioxidant Activity (CAA) Assay. (*) indicates significant difference with TBP; (#) indicates significant difference with tBHP-stimulated HepG2 cells at *p* = 0.05. Values presented as ± SEM from three independent trials with three replicates.

We tested how modifications on amino acid residues, length, hydrophobicity, and isoelectric point of TBP (VKAGFAWTANQQLS) affect its antioxidant activity using ABTS radical scavenging and cellular antioxidant activity assay. TBP exerted more than 50% radical scavenging activity (Figure 1a). Out of the 32 TBP analogs, 5 had increased activities while 20 were statistically comparable to the original peptide. Isoleucine-substituted analogs (3,5,6,8,10) and those with tryptophan replaced with glycine (12,13) significantly performed less. TBP analogs with terminal tryptophan were 1.5-fold better than TBP (Figure 1b). While no significant improvements were observed in TBP analogs compared to the parent peptide using cellular antioxidant activity (CAA) assay, seven test peptides were observed to exert cellular antioxidant activity in the organic peroxide (tBHP)-stimulated HepG2 cells (Figure 1c). The synthetic TBP used in this study was shown to be an active antioxidant in both ABTS radical scavenging and CAA assays along with peptides 2, 7, 15, and 17.

### 2.2. Peptide Modification Affects Bioactivity and Stability during in Silico Digestion

In silico digestion of TBP and its analogs shows that 24 TBP analogs and the original peptide can give rise to at least one antioxidant dipeptide (AW) while Peptide 29 can give rise to two antioxidant dipeptides (AW and TW) (Table 1) [51]. Peptide modification affected the virtual hydrolysablility of the analogs, with a drastic increase for Peptide 9 and a decline for Peptide 8, as reflected in the number of their generated fragments. In both cases, no antioxidant and ACE inhibitors were generated from these two peptides. Other than antioxidant peptides, TBP and its analogs can give rise to ACE and DPP-IV inhibitors after in silico digestion. Peptides 25, 26, and 29 had three DPP-IV inhibitory dipeptides (TW, VK, and TR) [52] while ACE inhibitory dipeptides (VK and AW) [51,53] were observed in more than half of the total digested test peptides. It is worth noting that most of the detected bioactive fragments were multifunctional BAPs.

### 2.3. Bioactivity Prediction for TBP and Its Analogs

Since we observed that TBP and its analogs can generate multifunctional dipeptides after in silico digestion, we tested if undigested peptides could exert other bioactivities as well. TBP analogs with Peptide Ranker scores >0.5 were predicted to be high confidence anti-inflammatory peptides, with Peptides 16, 29, and 31 being multifunctional (Table 2). Peptide 15, which also meets the bioactivity prediction threshold, shows potential multifunctionality as an antioxidant (Figure 1c) and anti-inflammatory peptide. It is worth noting that while Peptide 9 had a very low Peptide Ranker score, it had three predicted bioactivities which include anti-inflammatory, anti-angiogenic, and anti-hypertensive.

### 2.4. Applicability of Predicted Multifunctional BAPs

All five TBP analogs were predicted to be stable in both the intestine-like environment and plasma (Table 3). Half-lives of 16 amino acid residue ovalbumin peptides in the murine intestinal fluid were 0.00008–6.42 s [54]. The predicted plasma half-lives of our test peptides were 3 folds higher than that of insulin (4–6 min) [55]. These peptides were predicted to be non-toxic based on the presence/absence and position of specific residues like Cysteine, Histidine, Asparagine, and Proline, which are often present in toxic peptides [56]. Except for Peptide 9, all four TBP analogs were predicted to non-bitter based on their amino acid sequences, without any dependence on their functional domain or structural information [57]. 

## 3. Discussion

Since its report in 2007, not much has been done to further the research work on TBP. Considering the amount of tuna industry waste generated just from the backbone alone, the recovery of bioactive compounds from this material can not only help the waste problem but also improve resource utilization by the production of high-value products that have potential for pharmaceutical application [58,59]. As a seafood by-product BAP, TBP is of particular interest as it has an almost even distribution of aromatic (F,W), aliphatic non-polar (A,G,V,L), positively charged (K), and uncharged (T,S,Q,N) amino acid residues. Since amino acid composition and position are major determinants for activity and stability [3,60], we explored how peptide modification can affect the antioxidant property, stability, potential multifunctionality, and applicability of TBP and its analogs. 

Peptides 9, 14, 15, 21, and 33 displayed an increase in ABTS scavenging activity relative to TBP. Peptide 9 is a fully hydrophilic peptide displaying a significant increase in activity likely due to the enhanced solubility while the improvement in Peptides 14 and 15 with single tryptophan substitution on terminal ends could be attributed to the aromatic rings stabilizing electron transfer between the ABTS free radical and the antioxidant peptide. Peptides 3, 6, 8, and 10 had a significant drop in the activity which can be due to the decrease in solubility with the hydrophobic residue substitution, while removal of aromatic residues in Peptides 12 and 13 may have caused the decline in the activity. This confirms the cost-benefit balance of aromatic residues in antioxidant peptides wherein the aromatic rings may aid in the redox reactions but may also decrease peptide solubility in water. Tryptophan in myoglobin was a major driver for its antioxidant protection ratio while tyrosine position is critical for peroxynitrite protection [61]. 

While there was an observed improvement in the ABTS radical scavenging assay in peptides with terminal hydrophobic residues (Figure 1a,b) consistent with the literature [62,63,64], the enhanced activity in longer and more hydrophilic peptides was unexpected. Usually, peptides with low molecular weight, hydrophobic and aromatic acid in their structure have better antioxidant activities. Furthermore, these antioxidant activity improvements (Figure 1b) were not observed in the cell-based antioxidant assay using peroxide-stimulated, fluorescein-stained liver cells (Figure 1c). In fact, only Peptides 2, 7, 15, 17, and TBP showed consistent antioxidant property in both tests. All have hydrophobic amino acid residues (V,W,I) on terminal ends. The lack of concordance in radical scavenging and cell-based antioxidant assays has been a long-recognized challenge in antioxidants research as this limits the translatability of the work in a physiological context. It is for this reason that both should be undertaken during screening, with the cellular model given higher value as this will have closer resemblance in the actual condition where the bioactivity must be observed. It is of value to point out that the TBP analogs used in this study had lower purity (≥50%) than the parent peptide (>98%). While this can greatly affect how the analogs compare against TBP, their performance against other analogs can be compared reasonably. Our findings hold promise such that despite lower purity, some analogs still performed as antioxidant peptides with ABTS and CAA assays (Figure 1a,c). To effectively test how our modifications may have improved the antioxidant property, higher purity analogs must be used alongside parent peptide. A dose-response work using purer and wider concentration range can help elucidate essential modifications for potent antioxidant peptide preparation. 

For food-derived BAPs to have biomedical value, they must survive proteolytic conditions in the gut and be readily absorbed such that they can exert their desired biological activity in the biological system in vivo. The five consistent antioxidant peptides in this study were able to generate one antioxidant dipeptide (AW) [51] after in silico digestion using BIOPEP (Table 1). While digestion is considered a major bottleneck for most therapeutic peptide drug candidates, it is worth noting that it should not be viewed as all bad. After all, ingestion of protein precursors may generate bioactive peptide fragments in the gut after digestive enzymes cleave peptide bonds and liberate BAPs, enabling transport and absorption. Peptide 29 did not perform well as an antioxidant peptide candidate but after digesting it with pepsin, trypsin, and chymotrypsin using BIOPEP enzyme action tool [49], it generated (2) antioxidant, (2) ACE-inhibitory, and (3) DPPIV inhibitory dipeptides (Table 1). Our peptide modification affected the extent of peptide hydrolysablility in silico, as reflected in the number of generated fragments and degree of hydrolysis. While a higher number of fragments may suggest a higher chance of BAP generation, this was not true as can be observed in Peptide 9. The reoccurring bioactive dipeptides observed in this study include TR, AW, VK, and TW. Dipeptide VK was reported to exert ACE [53] and DPPIV [52] inhibition while AW and TW were both antioxidant and DPPIV inhibitors [51,52]. These dipeptides can cross intestinal membrane in their intact forms through passive (paracellular and transcellular diffusion) and active (transporter and transcytosis) transport [65,66]. When present in the bloodstream and can resist further degradation, considerable concentrations of these BAPs can elicit biological activity in vivo. Peptidomics and metabolomics studies would be needed to help establish the bioefficacy of BAPs.

Multifunctional BAPs are considered as therapeutic options for lifestyle-related diseases [3,8,50]. Since numerous pathologies are targeted to address an array of health complications, the use of multi-acting, potent, specific, stable, non-toxic, and non-bitter BAPs for functional food development is expanding. Here, we used multiple in silico platforms to screen for numerous bioactivities. We found five multifunctional peptides (Table 2) that have promising antioxidant, anti-inflammatory, antiangiogenic, antidiabetic, and antihypertensive potential. While Peptide Ranker has been repeatedly used in numerous BAP work for bioactivity potential assessment, we can see poor predictability for the scoring and our range of bioactivity screens. Peptides reaching the preset bioactivity threshold in the webserver (>0.5) showed specific bioactivities and multifunctionality. However, Peptide 9, which obtained a very low Peptide Ranker score, was predicted to be anti-inflammatory, anti-angiogenic, and anti-hypertensive. This can be due to the fact that the datasets (17,532 peptides) used for the machine learning/training included only cytokine and growth factors, hormones, antimicrobial, toxin/venom, anticancer peptides, and antifreeze proteins [67]. While in silico platforms significantly reduce the time and expenses involved in BAP screening, it is crucial to keep in mind that its predictive power is based on the training sets and databases used. The screening for anti-inflammatory peptides used PreAIP [68], which is a computational prediction platform trained to assess potential anti-inflammatory activity based on their primary sequence and structural information using a random forest classifier. The system was trained using anti-inflammatory peptides (positive samples) documented to induce IL-10, IL-4, IL-13, IL-22, TGF-β, and IFN-α/β in murine and human T-cell analyses. Our antidiabetic peptide screen used iDPPIV-SCM [69] that predicts peptides’ ability to inhibit DPP-IV using sequence information. By using a scoring card method (SCM), users are given a friendly interface to screen and shortlist potential DPP-IV inhibitors based on their composition and amino acid propensity scores. These researchers report that proline, tryptophan, methionine, and glutamine were abundant in DPPIV inhibitory peptides [69]. We used AntiAngioPred [70] to screen for the antiangiogenic potential of our 33 test peptides. This model predicts bioactivity potential based on amino acid residues and their position, being most antiangiogenic peptides have serine, proline, tryptophan, and cysteine in the N-terminal region while cysteine, glycine, and arginine are often found in the C-terminal region. Lastly, our antihypertensive screen used AHTPIN [71] that accounts for peptide length and amino acid residues in the prediction. Developers report that tryptophan, tyrosine, and proline are abundant in antihypertensive peptides.

To test whether the five multifunctional TBP analogs hold potential for further testing and application, we assessed for stability in the gut and the blood. Since BAPs have to survive digestive proteolysis and further degradation in the blood so target bioactivities can be exerted, we used HLP [72] to predict peptide stability in an intestine-like environment and PlifePred to predict their plasma stability. The half-lives of therapeutic peptides dictate bioavailability and distribution in the system, so it is important that we focus drug-design strategies on improving both potency and stability. Table 3 shows that our peptides were relatively stable in the gut compared to ovalbumin peptides digested with crude murine intestinal fluid [54,72]. HLP used datasets that calculated half-lives of peptides in undiluted crude intestinal proteolytic solution (mouse lavage), with some values extrapolated to account for dilution, they observed that large aromatic amino acid residues like phenylalanine, tyrosine, and tryptophan decrease peptide half-lives. The predicted multifunctional Peptide 9 was shown to be stable using HLP, while BIOPEP in silico digestion using pepsin, trypsin, and chymotrypsin showed that it was 76.92% hydrolyzed (Table 1 and Table 3). HLP developers state that their platform is not suitable for predicting the stability of modified peptides like N-terminal/C-terminal modifications. However, it also worth noting that in proteolysis simulation using BIOPEP, it is assumed that all peptide bonds are susceptible to hydrolysis which is often not the case in real experiments. On this, predictive data provided by both platforms can be revisited using simulated digestion models.

With PlifePred [73], all five peptides were predicted to be more stable than insulin [55]. Their dataset included structurally annotated peptides having >5–50 amino acid residues, with half-lives >20 s to <24 h in blood, urine, intestine, kidney, brain homogenates, and various cell culture media, all free of complex terminal modifications. Using these, they reported that negatively charged (Glu) and small-sized residues (Ala, Glu, Ile, and Leu) were frequently observed in peptides with long half-lives while less stable peptides are rich with aromatic (Tyr and Phe) and neutral amino acids (Gly, His, Ser, and Tyr) [73]. Our multifunctional peptides were predicted to be non-toxic based on ToxinPred [56] which observed the abundance of cysteine, histidine, asparagine, and proline in specific positions in toxic peptides. Only one of the multifunctional peptides was predicted to be non-bitter with iBitter-SCM [57]. Since most of the BAP-based commercial products are consumed through ingestion [10,74], it is important to account for the sensory experience target consumers may have in functional food product development. This sequence-based predictor calculates for bitter scores based on the propensity scores of amino acid residues (Gly, Phe, Pro, Glu, and Asp) in bitter peptides [57].

Here we show that complementing in vitro screening with in silico testing can not only increase the number of bioactivity testing done but also provide an opportunity to narrow down BAP prospects with the use of predictive platforms for stability, toxicity, and bitterness. In the big data era, the use of the bioinformatics-based approach help saves a significant amount of resources while casting a bigger net for prospect BAPS. This is of particular importance for BAPs prepared with simulated digestion and those identified using de novo approaches. Since validation is crucial, using in silico platforms makes the drug candidate shortlist much faster and cheaper making this peptide drug discovery approach more efficient.

## 4. Materials and Methods

### 4.1. Peptide Analog Selection

The 32 modified TBP analogs were selected based on the earlier research works identifying residues modulating antioxidant activity. Hydrophobic residues were shown to be effective against the peroxyl radical [61], therefore six analogs of TBP (Peptides 2–7) were developed in which a single hydrophilic residue of TBP was substituted with a hydrophobic isoleucine residue. Additionally, analogs 8–10 were created with either only hydrophobic or hydrophilic residues to identify how large-scale substitutions can affect peptide solubility, free radical scavenging, and cell-penetrating ability. Peptides 11–16 were developed by modifying the aromaticity of TBP as it was shown that aromatic residues contributed to improved antioxidant, ACE-inhibitory, and renin-inhibitory activity [75]. In Analogs 11–13, the aromatic residues (F5 and W7) were removed and replaced with glycine given its small and non-interfering side chain. Analogs 14–16 had tryptophan(s) added to a terminal end. While TBP has aromatic residues within its sequence, tryptophan was added to the C and N terminus as the location of aromatic residues has been shown to modulate antioxidant activity [76]. Specifically, tryptophan was chosen as the aromatic residue as it has been shown to act as an antioxidant individually and has hydrogen donating capabilities [77]. Acidic residues were reported to be effective against the peroxynitrite [61], therefore four analogs (Peptides 17–20) were developed with basic residues within TBP replaced with acidic residues. Smaller antioxidant peptides (<1 kDa) were shown to have higher antioxidant activity [78]. Analogs 21–24 were developed to increase the peptide length of TBP through the addition of glycine residues to understand whether peptide length is inversely related to antioxidant activity. Analogs 25–28 were developed to adjust the isoelectric point (pI) of TBP. Given the charged nature of certain radicals such as ABTS, we sought to understand whether the modification of peptide pI would change affinity with the radical and solubility in water. Analog 29 had the two smallest residues (G4 and A8) removed and replaced with tryptophan to understand the effect of steric hindrance on antioxidant activity. Analog 30 had the serine in TBP removed since L-serine has shown to have antioxidant effects and it was hypothesized that it also plays a significant role in the activity of TBP [79]. Analog 31 had cysteine caps added onto TBP as cysteine is a powerful antioxidant due to its sulfhydryl group and can form disulfide linkages to change peptide folding and possible dimerization [80]. Analogs 32 and 33 were developed to disrupt the secondary structure of TBP through the substitution of proline residues into the peptide, as it is a common disrupter of secondary structure [81]. Table 4 shows the sequences and modifications of the peptide analogs selected.

### 4.2. Materials

TBP and its analogs (2–33) were obtained from Thermo Fisher Scientific through LifeTech (Carlsbad, CA, USA). TBP was synthesized at 98% purity while analogs were synthesized at ≥50% purity. All peptides were resuspended in phosphate-buffered saline (PBS) at pH 7 and stored at −20 ℃ until use. ABTS (2,2’-azino-bis 3-ethylbenzothiazoline-6-sulfonic acid), potassium persulfate, α-tocopherol, Trolox, DCFDA (2′,7′-dichlorofluorescin diacetate), and tBHP (tert-butyl hydroperoxide) were purchased from Sigma-Aldrich (St. Louis, MO, USA). Hepatocellular carcinoma (HepG2) cells were obtained from ATCC (Manassas, VA, USA). Dulbecco Modified Eagle’s Medium (DMEM) and penicillin-streptomycin have been obtained from Thermo Fisher Scientific (Waltham, MA, USA) while Fetal Bovine Serum (FBS) was procured from R&D Systems (Minneapolis, MN, USA).

### 4.3. Antioxidant Assays

Stable radical scavenging activity was tested using ABTS [82]. Briefly, ABTS working solution was prepared from a 12–14 h old solution of 5 mL 7 mM ABTS and 88 µL 140 mM Potassium persulfate. This was diluted with methanol or buffered saline solution to reach an absorbance of 0.70 ± 0.05 at 734 nm. For peptide screening, 10 µL peptide solutions (2 mg/mL) or antioxidant control (1 µL/mL α-tocopherol) are seeded in a clear 96-well plate, followed by 190 µL ABTS solution. After a 10 min incubation, absorbance reading was obtained using Spectramax (Molecular Devices).

$$\%\;ABTS\;Radical\;Scavenging=\frac{\left(\mathrm{Abs}\;\mathrm{ABTS}+\mathrm{Vehicle}\right)-\left(\mathrm{Abs}\;\mathrm{ABTS}+\mathrm{Sample}\right)}{\left(\mathrm{Abs}\;\mathrm{ABTS}+\mathrm{Vehicle}\right)}\times 100$$

To evaluate the translatability of antioxidant performance in a physiological setting, a cellular antioxidant assay was conducted [83]. Confluent HepG2 culture was harvested and seeded in black 96-well plates at 6 × 10^4^ per well. After overnight incubation, cells were treated with 25 µM DCFDA dissolved in DMEM with 10% FBS and 1% penicillin-streptomycin. After staining, cells were then treated with the 10 µL peptides (2 mg/mL) and antioxidant controls. All wells were challenged 500 µM tBHP. After 1-h incubation, fluorescence reading at excitation 485 nm and emission 538 nm was obtained using Spectramax (Molecular Devices).

$$\%\;Control=\frac{\left(\mathrm{RFU}\;\mathrm{tBHP}\;\mathrm{challenged}+\mathrm{Sample}\right)}{\left(\mathrm{RFU}\;\mathrm{tBHP}\;\mathrm{challenged}+\mathrm{Vehicle}\right)}\times 100$$

### 4.4. In Silico Digestion

To determine whether TBP and its analogs can still exert bioactivity after ingestion, we conducted an in silico analysis using BIOPEP [49]. To simulate digestion, we subjected the peptide sequences to a virtual hydrolytic breakdown using pepsin (pH 1.3), trypsin, and chymotrypsin. With the known cleavage sites for each enzyme, BIOPEP generates a theoretical value for the degree of hydrolysis based on the number of hydrolyzed peptide bonds against the total peptide bonds in the sample. After digestion, peptide fragments released with the enzyme action are used against bioactive peptide databases to test whether known BAPs were generated. The relative frequency of a bioactive fragment release after enzyme treatment (A_E_) is provided based on specific bioactivity. This value is calculated as

$$AE=\frac{\left(\mathrm{Number}\;\mathrm{of}\;\mathrm{Bioactive}\;\mathrm{Fragments}\right)}{\left(\mathrm{Number}\;\mathrm{of}\;\mathrm{Amino}\;\mathrm{Acid}\;\mathrm{Residues}\right)}$$

### 4.5. Multifunctionality, Stability, Toxicity and Bitterness Prediction 

To assess the potential of TBP and its analogs to be multifunctional, we used multiple bioinformatics-based platforms for bioactivity prediction. Using a novel neural network Peptide Ranker [67], peptide sequences were rank-scored based on their probability to be bioactive. Bioactivity prediction for anti-inflammatory, antidiabetic, antiangiogenic, and antihypertensive potential were done using online web servers namely PreAIP [71], iDPPIV-SCM [69], AntiAngioPred [70], and AHTPIN [84]. To investigate whether the predicted multifunctional TBP analogs would be promising test peptides for therapeutic application, we predicted their stability in an intestine-like environment and the blood using HLP [72] and PlifePred [73], respectively. We also assessed whether these peptides can exert some toxicity and elicit aversion due to bitterness using online platforms ToxinPred [56] and iBitter-SCM [57]. Table 5 provides a summary of *in silico* platforms used.

## 5. Conclusions

Seafood by-product such as tuna backbone is a promising source of multifunctional bioactive peptides that have therapeutic potential. Some TBP analogs, though of lesser purity, performed well in our antioxidant assays, which merits further research attention utilizing more highly purified peptides. With an in silico approach, bioactivity prospecting and screening were conducted in a much shorter period with significantly less cost. Using TBP and its analogs, we show that five multifunctional peptides can be used to the next level of testing for bioactivity and peptide drug design simulations. Overall, our study demonstrates that coupling in vitro and in silico approaches is an effective strategy to accelerate BAP drug discovery.

## Figures and Tables

**Table 1:** Predictive degree of hydrolysis and frequency of bioactive peptide fragment generation after in silico digestion of TBP and its analogs using BIOPEP ^a^.

Peptide	% Degree of Hydrolysis	AA Residue	Fragments	Antioxidant		ACE-1 Inhibition		DPP-IV Inhibition	
				A_E_	No. of BAPs	A_E_	No. of BAPs	A_E_	No. of BAPs
TBP	38.46	14	6	0.0714	1	0.1429	2	0.1429	2
2	28.57	15	5	0.0667	1	0.0667	1	0.0667	1
3	28.57	15	5	__		0.0667	1	0.0667	1
4	35.71	15	6	0.0667	1	0.1333	2	0.1333	2
5	28.57	15	5	0.0667	1	0.0667	1	0.0667	1
6	38.46	15	6	0.0714	1	0.1429	2	0.1429	2
7	35.71	15	6	0.0667	1	0.1333	2	0.1333	2
8	14.29	15	3	__	__	__	__	__	
9	76.92	14	11	__	__	__	__	0.0714	1
10	28.57	8	2	__	__	__	__	__	
11	30.77	14	5	__	__	0.0714	1	0.0714	1
12	28.57	15	5	__	__	0.0667	1	0.0667	1
13	23.08	15	4	__	__	0.0714	1	0.0714	1
14	46.15	14	7	0.0714	1	0.0714	1	0.0714	1
15	38.46	15	6	0.0714	1	0.1429	2	0.1429	2
16	46.15	14	7	0.0714	1	0.0714	1	0.0714	1
17	30.76	14	5	0.0714	1	0.0714	1	0.0714	1
18	38.46	14	6	0.0714	1	0.1429	2	0.1429	2
19	30.76	14	5	0.0714	1	0.0714	1	0.0714	1
20	30.76	14	5	0.0714	1	0.0714	1	0.0714	1
21	35.71	16	6	0.0667	1	0.1333	2	0.1333	2
22	29.41	18	6	0.0556	1	0.1111	2	0.1111	2
23	26.32	20	6	0.0500	1	0.1000	2	0.1000	2
24	22.22	19	5	0.0526	1	0.1053	2	0.1053	2
25	46.66	17	8	0.0625	1	0.1250	2	0.1875	3
26	42.85	16	7	0.0667	1	0.1333	2	0.2000	3
27	33.33	16	6	0.0625	1	0.1250	2	0.1250	2
28	28.57	16	5	0.0667	1	0.0667	1	0.0667	1
29	53.84	14	8	0.1429	2	0.1429	2	0.2143	3
30	38.46	14	5	0.0714	1	0.1429	2	0.1429	2
31	31.25	17	6	0.0588	1	0.0588	1	0.0588	1
32	30.76	15	5	0.0714	1	0.0714	1	0.0714	1
33	30.76	15	5	0.0714	1	0.0714	1	0.0714	1

^a^http://www.uwm.edu.pl/biochemia/index.php/en/biopep. (accessed on 2 February 2021) BAP, bioactive peptides.

**Table 2:** Bioactivity prediction for TBP and its analogs.

Peptides	Peptide Ranker ^a^	Anti-Inflammatory ^b^		Antidiabetic ^c^		Anti-Angiogenic ^d^		Antihypertensive ^e^	
	Score	Score	Descriptor	Score	Descriptor	Score	Descriptor	Score	Descriptor
TBP	0.250852	0.403	Medium Confidence AIP	257.92	non-DPPIV	−0.96	Non-anti-angiogenic	−1.49	Non-AHT
2	0.282936	0.424	Medium Confidence AIP	275.54	non-DPPIV	−0.64	Non-anti-angiogenic	−1.66	Non-AHT
3	0.146123	0.366	Low Confidence AIP	243.08	non-DPPIV	−1.18	Non-anti-angiogenic	−1.89	Non-AHT
4	0.318239	0.435	Medium Confidence AIP	254.08	non-DPPIV	−0.62	Non-anti-angiogenic	−1.87	Non-AHT
5	0.301595	0.412	Medium Confidence AIP	252.77	non-DPPIV	−0.52	Non-anti-angiogenic	−1.6	Non-AHT
6	0.341132	0.415	Medium Confidence AIP	250.92	non-DPPIV	−0.98	Non-anti-angiogenic	−2.18	Non-AHT
7	0.29307	0.43	Medium Confidence AIP	271.15	non-DPPIV	−1.15	Non-anti-angiogenic	−1.96	Non-AHT
8	0.581973	0.611	High Confidence AIP	250.92	non-DPPIV	−0.57	Non-anti-angiogenic	−0.1	Non-AHT
9	0.0930615	0.607	High Confidence AIP	156.15	non-DPPIV	1.8	Anti-angiogenic	0.04	AHT
10	0.38964	0.334	negative AIP	276.50	non-DPPIV	−1.25	Non-anti-angiogenic	−1.43	Non-AHT
11	0.21006	0.402	Medium Confidence AIP	250.15	non-DPPIV	−1.02	Non-anti-angiogenic	−1.42	Non-AHT
12	0.146824	0.359	Low Confidence AIP	238.46	non-DPPIV	−1.68	Non-anti-angiogenic	−1.62	Non-AHT
13	0.120407	0.362	Low Confidence AIP	230.69	non-DPPIV	−1.52	Non-anti-angiogenic	−1.34	Non-AHT
14	0.437794	0.421	Medium Confidence AIP	268.00	non-DPPIV	−0.29	Non-anti-angiogenic	−1.15	Non-AHT
15	0.539862	0.477	High Confidence AIP	286.00	non-DPPIV	−0.83	Non-anti-angiogenic	−1.41	Non-AHT
16	0.751235	0.493	High Confidence AIP	296.08	DPPIV	−0.11	Non-anti-angiogenic	−0.95	Non-AHT
17	0.322171	0.403	Medium Confidence AIP	273.38	non-DPPIV	−0.96	Non-anti-angiogenic	−1.57	Non-AHT
18	0.225945	0.416	Medium Confidence AIP	248.77	non-DPPIV	−1.44	Non-anti-angiogenic	−2.16	Non-AHT
19	0.279449	0.419	Medium Confidence AIP	264.23	non-DPPIV	−1.29	Non-anti-angiogenic	−1.96	Non-AHT
20	0.280885	0.404	Medium Confidence AIP	255.08	non-DPPIV	−1.43	Non-anti-angiogenic	−1.89	Non-AHT
21	0.279711	0.231	negative AIP	251.57	non-DPPIV	−1.23	Non-anti-angiogenic	−1.62	Non-AHT
22	0.384437	0.512	High Confidence AIP	241.25	non-DPPIV	−1.37	Non-anti-angiogenic	−1.46	Non-AHT
23	0.492159	0.476	High Confidence AIP	233.22	non-DPPIV	−1.15	Non-anti-angiogenic	−1.02	Non-AHT
24	0.477331	0.441	Medium Confidence AIP	233.22	non-DPPIV	−1.11	Non-anti-angiogenic	−0.22	Non-AHT
25	0.255879	0.524	High Confidence AIP	229.13	non-DPPIV	−0.64	Non-anti-angiogenic	−1.82	Non-AHT
26	0.131233	0.311	negative AIP	232.21	non-DPPIV	−0.29	Non-anti-angiogenic	−1.43	Non-AHT
27	0.194823	0.244	negative AIP	253.86	non-DPPIV	−1.21	Non-anti-angiogenic	−1.8	Non-AHT
28	0.227495	0.245	negative AIP	268.21	non-DPPIV	−1.09	Non-anti-angiogenic	−1.62	Non-AHT
29	0.509724	0.541	High Confidence AIP	299.67	DPPIV	1.01	Anti-angiogenic	−0.31	Non-AHT
30	0.310984	0.423	Medium Confidence AIP	274.67	non-DPPIV	−1.35	Non-anti-angiogenic	−1.6	Non-AHT
31	0.598761	0.57	High Confidence AIP	231.27	non-DPPIV	0.64	Anti-angiogenic	−1.19	Non-AHT
32	0.345985	0.388	Low Confidence AIP	294.85	DPPIV	−0.68	Non-anti-angiogenic	−0.68	Non-AHT
33	0.444989	0.408	Medium Confidence AIP	307.15	DPPIV	−0.6	Non-anti-angiogenic	−0.09	Non-AHT

^a^http://distilldeep.ucd.ie/PeptideRanker/ (accessed on 2 February 2021). ^b^ http://kurata14.bio.kyutech.ac.jp/PreAIP/ (accessed on 2 February 2021). ^c^ http://camt.pythonanywhere.com/iDPPIV-SCM (accessed on 2 February 2021). ^d^ https://webs.iiitd.edu.in/raghava/antiangiopred/ (accessed on 2 February 2021). ^e^ http://crdd.osdd.net/raghava/ahtpin/ (accessed on 2 February 2021).

**Table 3:** Predicted stability, toxicity, and bitterness for multifunctional TBP analogs.

TBP Analogs	Activities	Half-Life (Sec) Intestine ^a^	Half-Life (Sec) Blood ^b^	Toxicity ^c^ Score Descriptor		Bitterness ^d^ Score Descriptor	
RKKRKRWTKNQQRS	AI, AG, AH	2.021	967.61	−1.41	Non-Toxin	279.23	non-Bitter
VKAGFAWTANQQLW	AO, AI	2.847	1099.41	−1.32	Non-Toxin	398.38	Bitter
WKAGFAWTANQQLW	AI, DI	2.873	1004.21	−1.23	Non-Toxin	396.46	Bitter
VKAWFWTWNQQLS	AI, DI, AG	2.834	1176.91	−1.42	Non-Toxin	396.92	Bitter
CVKAGFAWTANQQLSC	AI, AG	2.809	931.61	−1.09	Non-Toxin	397.53	Bitter

AI, anti-inflammatory; AG, anti-angiogenic; AH, antihypertensive; AO, antioxidant; DI, DPPIV Inhibition. ^a^ http://crdd.osdd.net/raghava/hlp/ (accessed on 2 February 2021). ^b^ https://webs.iiitd.edu.in/raghava/plifepred/ (accessed on 2 February 2021). ^c^ https://webs.iiitd.edu.in/raghava/toxinpred (accessed on 2 February 2021). ^d^ http://camt.pythonanywhere.com/iBitter-SCM (accessed on 2 February 2021).

**Table 4:** Tuna backbone peptide and its analogs.

	Peptide Sequence	Modifications from TBP	Mol. Weight (g/mol)	Isoelectric Point
TBP	VKAGFAWTANQQLS		1520.71	10.1
2	VIAGFAWTANQQLS	K(2) → I(2)	1505.69	6
3	VKAGFAITANQQLS	W(7) → I(7)	1447.66	10.1
4	VKAGFAWIANQQLS	T(8) → I(8)	1532.76	10.1
5	VKAGFAWTAIQQLS	N(10) → I(10)	1519.76	10.1
6	VKAGFAWTANIQLS	Q(11) → I(11)	1505.74	10.1
7	VKAGFAWTANQQLI	S(14) → I(14)	1546.79	10.1
8	VIAGFAIIAIIILI	Full Hydrophobic Peptide	1439.89	6
9	RKKRKRWTKNQQRS	Full Hydrophilic Peptide	1900.22	13
10	VAGFAAL	Full Hydrophobic Peptide	647.77	6
11	VKAGGAWTANQQLS	F(5) → G(5)	1430.58	10.1
12	VKAGFAGTANQQLS	W(7) → G(7)	1391.55	10.1
13	VKAGGAGTANQQLS	F(5), W(7) → G(5,7)	1301.42	10.1
14	WKAGFAWTANQQLS	V(1) → W(1)	1607.79	10.1
15	VKAGFAWTANQQLW	S(14) → W(14)	1619.84	10.1
16	WKAGFAWTANQQLW	V(1), S(14) → W(1,14)	1706.92	10.1
17	VDAGFAWTANQQLS	K(2) → D(2)	1507.62	3.1
18	VKAGFAWTANDQLS	Q(11) → D(11)	1507.67	6.8
19	VDAGFAWTANDQLS	K(2), Q(11) → D(2,11)	1494.58	2.9
20	VDAGFAWTANDDLS	K(2), Q(11), Q(12) → D(2,11,12)	1481.54	2.8
21	VKAGFAWGTANQQLS	G(8) inserted	1577.76	10.1
22	VKAGFAWGGGTANQQLS	G(8,9,10) inserted	1691.86	10.1
23	VKAGGGFAWGGGTANQQLS	G(5,6,10,11,12) inserted	1805.97	10.1
24	VKAGGGFAWGGGTANGGQQ	G(5,6,10,11,12,16,17) inserted, LS removed	1719.83	10.1
25	VKAGFAWTRKANQQLS	R(9), K(10) inserted	1805.07	11.7
26	VKAGRAWTRANQQLS	R(5), R(10) inserted	1685.91	12.5
27	VKAGFAWTDANQQLS	D(9) inserted	1635.8	6.8
28	VDAGFAWTDANQQLS	D(9) inserted, K(2) → D(2)	1622.71	2.9
29	VKAWFWTWNQQLS	G(4), A(8) → W(4,8)	1693.93	10.1
30	VKAGFAWTANQQL	S(14) removed	1433.63	10.1
31	CVKAGFAWTANQQLSC	C(1,16) inserted	1727	8.3
32	VPAGFAWTANQQLS	K(2) → P(2)	1489.65	6
33	VPAGFAWTANQPLS	K(2), Q(12) → P(2,12)	1458.64	6

**Table 5:** In Silico scoring and screening platforms for bioactive peptides.

Platform/Server	Predictive Purpose	Link
Peptide Ranker	Bioactivity Potential Scoring	http://distilldeep.ucd.ie/PeptideRanker (accessed on 14 January 2021)
PreAIP	Anti-inflammatory Peptide Screening	http://kurata14.bio.kyutech.ac.jp/PreAIP/ (accessed on 17 February 2021)
iDPPIV-SCM	DPPIV Inhibitor Peptide Screening	http://camt.pythonanywhere.com/iDPPIV-SCM (accessed on 17 February 2021)
AntiAngioPred	Anti-angiogenic Peptide Screening	https://webs.iiitd.edu.in/raghava/antiangiopred/ (accessed on 17 February 2021)
AHTPIN	Antihypertensive Peptide Screening	http://crdd.osdd.net/raghava/ahtpin/ (accessed on 17 February 2021)
HLP	Intestinal Stability	http://crdd.osdd.net/raghava/hlp/ (accessed on 21 February 2021)
PlifePred	Plasma Stability	https://webs.iiitd.edu.in/raghava/plifepred/ (accessed on 21 February 2021)
ToxinPred	Toxicity Screening	https://webs.iiitd.edu.in/raghava/toxinpred (accessed on 21 February 2021)
iBitter-SCM	Bitterness Peptide Screening	http://camt.pythonanywhere.com/iBitter-SCM (accessed on 21 February 2021)

## Data Availability

Not applicable.

## Sample Availability

Samples of the compounds are available from the authors.
